# Generating a Moderated Mediation Model of Positive Outcome Expectancy and Aggression

**DOI:** 10.3390/bs13090729

**Published:** 2023-08-31

**Authors:** Jiaming Wei, Ling-Xiang Xia

**Affiliations:** 1 Research Center of Psychology and Social Development, Faculty of Psychology, Southwest University, Chongqing 400715, China; wjm233@email.swu.edu.cn; 2 Key Laboratory of Cognition and Personality, Ministry of Education, Southwest University, Chongqing 400715, China

**Keywords:** outcome expectancy, aggression, moral disengagement, college students

## Abstract

According to previous theories of aggression, positive outcome expectancy for aggression can predict aggression, while moral disengagement and negative outcome expectancy for aggression may, respectively, serve as mediators and moderators in this prediction process. To test the hypothesis, Study 1 first developed the Aggression Outcome Expectancy Questionnaire and examined its two-factor structure, which consists of positive and negative outcome expectancy for aggression. Next, 677 college students were recruited to participate in Study 2 and were asked to complete the Aggression Outcome Expectancy Questionnaire, Civic Moral Disengagement Questionnaire, and Buss–Perry Aggression Questionnaire. The findings indicated the following: (1) The Aggression Outcome Expectancy Questionnaire for college students demonstrated acceptable reliability and construct validity, confirming the two-factor structure of aggression outcome expectancy. (2) After controlling for sex and age, moral disengagement partially mediated the relationship between positive outcome expectancy and aggression. (3) Negative outcome expectancy for aggression moderated the effect of positive outcome expectancy on aggression, as well as moral disengagement. Specifically, negative outcome expectancy for aggression attenuated the positive predictive effect of positive outcome expectancy on aggression and moral disengagement. In conclusion, the present study extends our understanding of the motivational mechanism of aggression, offering a theoretical reference for preventing and intervening in aggressive behavior among college students.

## 1. Introduction

Aggression refers to harmful behavioral responses or tendencies driven by the intention to cause harm [1], and it is regarded as a significant public health issue [2]. The occurrence of aggressive behaviors is pervasive among college students, and it may be associated with problems such as depression and suicidal inclination [2,3]. Gaining a deep understanding of the psychological mechanisms that foster aggression in college students is essential to implementing specific prevention and intervention measures. Hence, investigating the influencing factors and mechanisms of aggression in college students is of both theoretical significance and practical importance.

Cognitive factors play a critical role in the formation and development of aggression [4,5,6]. Among these factors, outcome expectancy is a widely recognized influential cognitive variable in aggression [4,5,7], representing the cognitive reactions or tendencies of anticipating the potential outcomes of engaging in behaviors that harm others [6,8,9]. Aggressive outcome expectancy can be conceptually divided into positive and negative dimensions based on the content and valence of outcomes [6,10,11,12]. Although some research indirectly supports this classification, direct empirical evidence is still insufficient [10,11,12]. Therefore, we assume that aggressive outcome expectancy has a two-factor structure, which consists of positive and negative aggressive outcome expectancies.

Positive outcome expectancy is believed to play a significant role in the acquisition, occurrence, and maintenance of aggression [5,7,13]. Research findings from various contexts, including school bullying [14], workplace aggression [12], and intimate partner violence [13,15], consistently indicate that positive outcome expectancy significantly predicts aggressive behavior. According to previous studies, we propose that positive outcome expectancy fosters aggression primarily due to two primary factors.

Firstly, positive outcome expectancy can provide a motivational foundation to engage in aggression. According to social learning theory [5], positive outcome expectancy provides a reinforcement-based motivation for aggression. Additionally, the social information processing model [6,16] suggests that anticipated positive outcomes serve as incentives for aggressive behavior. In other words, positive outcome expectancy provides goals and driving forces for aggression, enhancing individuals’ tendency to engage in aggression [6,17].

Secondly, positive outcome expectancy might contribute to aggression by influencing moral cognition related to aggression, particularly moral disengagement. Moral disengagement refers to the cognitive reconstruction of unethical behaviors as acceptable or moral, aimed at reducing or avoiding moral inhibitions and feelings of moral dissonance [18,19,20]. Positive outcome expectancy induces individuals to generate aggressive intentions that conflict with moral standards, leading to moral dissonance [17,19,21]. To cope with the mental discomfort arising from moral dissonance and alleviate the tension between aggressive intentions and moral norms, individuals may employ moral disengagement strategies [19,20]. In other words, the approach motivation elicited by positive outcome expectancy for aggression serves as the fundamental driving force for individuals to engage in moral disengagement. A cross-sectional study revealed a significant positive correlation between the positive outcome expectancy of bullying and moral disengagement [14]. In an experimental study, individuals displayed moral disengagement towards their harmful actions when they expected to acquire monetary benefits by causing harm to others [22]. On the other hand, according to the aggression motivation model, moral disengagement is an important cognitive factor that facilitates aggression by reducing or nullifying the inhibitory effects of the moral system on aggression [20,23]. Evidence from longitudinal studies supports that moral disengagement contributes to aggression [19,23,24]. For example, in a sample of college students, Cen et al. (2022) found that moral disengagement significantly predicted aggressive behavior six months later [23]. Therefore, based on the evidence from the above studies, we hypothesize that moral disengagement mediates the relationship between positive outcome expectancy and aggression. In particular, positive outcome expectancy for aggression may intensify individuals’ moral disengagement, subsequently facilitating aggression.

On the other hand, while some studies consistently find that positive outcome expectancy significantly predicts aggression [9,10,12,25,26], some researchers have found no significant relationship between the two [27,28]. This indicates that there might be moderating factors influencing the relationship between the two. Some researchers suggested that negative outcome expectancy could be an important moderating variable in the relationship between positive outcome expectancy and aggression. For example, according to the Response Evaluation and Decision model [7], the impact of positive outcome expectancy on aggression is influenced by negative outcome expectancy. Miles-McLean et al. (2021) propose that negative outcome expectancy may serve as a moderating variable, weakening the driving effect of positive outcome expectancy on aggression [13]. Moreover, the I^3^ theory also suggests that behavioral inhibitory factors (such as negative outcome expectancy) can moderate the relationship between driving factors (such as positive outcome expectancy) and behavior [29]. Negative outcome expectancy reflects the awareness of the potential risks of aggression, such as feelings of guilt, social reputation deterioration, and interpersonal relationship losses [9,11,30]. These factors influence individuals’ perception and experience of whether aggression is worthwhile. Hence, when negative outcome expectancy is high, it can weaken the aggressive proclivities engendered by positive outcome expectancy, resulting in a diminished driving force of positive outcome expectancy on aggression. In summary, both positive and negative outcome expectancies jointly influence aggression. Therefore, we propose the hypothesis that negative outcome expectancy weakens the promoting effect of positive outcome expectancy on aggression.

Furthermore, negative outcome expectancy may also moderate the relationship between positive outcome expectancy and moral disengagement. Specifically, under conditions of low negative outcome expectancy, individuals perceive lower levels of risk related to aggression, which leads them to believe that aggression is relatively worthwhile. Consequently, positive outcome expectancy can serve as a powerful driving force for moral disengagement, thereby enhancing the likelihood of moral disengagement. On the other hand, in conditions of high negative outcome expectancy, individuals perceive increased risks related to aggression, leading them to consider aggression as not worthwhile and unnecessary. In such a situation, the promoting effect of positive outcome expectancy on aggression intentions is limited. There is no apparent internal moral conflict, and therefore, there is no need for moral disengagement. Therefore, we propose the last hypothesis: negative outcome expectancy mitigates the relationship between positive outcome expectancy and moral disengagement.

**Figure 1:**
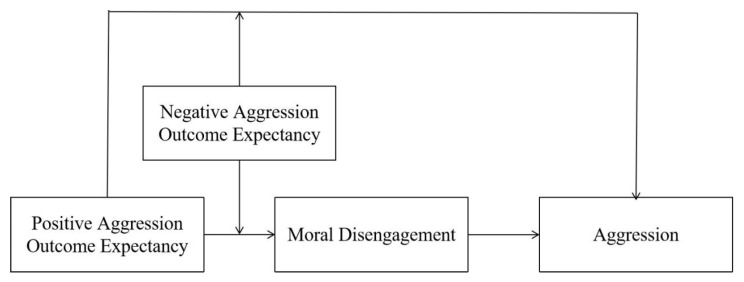
Proposed research model in Study 2.

Therefore, based on existing aggression theories (e.g., aggression motivation model, I^3^ theory) and empirical findings, we propose a mediation model with moral disengagement as the mediating variable and negative outcome expectancy as a moderator to examine the relationships between positive outcome expectancy, aggression, and moral disengagement (Figure 1 represents the hypothesized model of our study). Study 1 aimed to investigate the two-factor structure of aggression outcome expectancy (i.e., positive and negative aggression outcome expectancies). Hence, we intend to develop an aggression outcome expectancy questionnaire and recruit a group of college student participants to complete it. Following this, we will employ confirmatory factor analysis to validate the psychological structure of aggression outcome expectancy. In Study 2, we recruited a new group of college student participants and measured their levels of aggression, moral disengagement, and positive and negative outcome expectations of aggression with their informed consent. Subsequently, these data were utilized to examine the proposed moderated mediation model. In summary, this study aims to test the following Hypotheses 1–4 among college students:

**Hypothesis** **1.** *Aggression outcome expectancy comprises two factors: positive and negative outcome expectancies.*

**Hypothesis** **2.** *The relationship between positive aggression outcome expectancy and aggression is mediated by moral disengagement.*

**Hypothesis** **3.** *Negative aggression outcome expectancy, serving as a moderator, attenuates the positive predictive effect of positive aggression outcome expectancy on aggression.*

**Hypothesis** **4.** *Negative aggression outcome expectancy, serving as a moderator, attenuates the positive predictive effect of positive aggression outcome expectancy on moral disengagement.*

## 2. Study 1

### 2.1. Participants

In accordance with the guidance of prior studies [31,32], it is advisable to have a sample size of at least 200 when conducting factor analysis or a minimum of five times the number of questionnaire items. Therefore, in Study 1, with the aim of obtaining reliable results, we planned to recruit more than 300 participants.

A total of 352 questionnaires were distributed using a convenience sampling approach through a combination of online and offline methods. We recruited participants from three universities located in Chongqing, Jiangsu, and Shandong Province. Initially, we posted recruitment advertisements in online communities, promising the confidentiality of questionnaire data and offering compensation to participants contingent on their data meeting our quality criteria. Once participants agreed to participate in the study, we provided them with a web link that granted them access to the WenJuanXing platform, an online survey website in Chinese, where they could proceed to complete the questionnaire. After data collection, if the data from participants meets any of the following conditions, it will be excluded: (1) choosing the same response option for all (or almost all) items; (2) incomplete or multiple responses to a single item; and (3) completing the online questionnaire in an excessively short time. As a result, 331 valid questionnaires were retained, resulting in an effective response rate of 94.03%. Following the exclusion of invalid data, the sample consisted of 133 male participants (40.18%) and 198 female participants (59.82%). The age of the participants ranged from 17 to 23 years (*M*_age_ = 20.11, *SD*_age_ = 1.97). Once the questionnaire quality was confirmed, participants were provided with compensation.

### 2.2. Measurement

To address the targeted measurement of positive and negative aggression outcome expectancies among college students, the present study developed the Aggression Outcome Expectancy Questionnaire (AOEQ) based on previous work from our research team. The questionnaire was developed by referencing the aggression and illegal behavior subscales from the Cognitive Appraisal of Risky Events Questionnaire [33]. Following discussions with a psychology professor and several graduate students, revisions were made to the questionnaire items. The final version measured individuals’ positive and negative outcome expectancies related to 15 common aggressive behaviors (e.g., “Physically attacking others using tools or weapons”), including online aggression. There are 30 items in the questionnaire, which are categorized into two subscales: positive aggression outcome expectancy and negative aggression outcome expectancy. Participants were required to rate their “anticipated satisfaction” and “anticipated risk” regarding the aggressive behaviors presented in the questionnaire. “Anticipated satisfaction” referred to the overall degree to which the behavior was expected to yield satisfactory or desirable outcomes for oneself, while “anticipated risk” indicated the overall degree to which the behavior was expected to result in undesirable or unfavorable outcomes for oneself. The questionnaire utilized a 7-point Likert scale (ranging from 1, “extremely low”, to 7, “extremely high”) for scoring. Higher total mean scores indicated a greater degree of positive or negative aggression outcome expectancy.

### 2.3. Statistical Analysis

Statistical analyses were performed using both SPSS25 and Mplus7. Specifically, the questionnaire data will be subjected to item analysis, internal consistency reliability testing, and confirmatory factor analysis.

### 2.4. Results

Firstly, the scores of positive and negative aggression outcome expectancies were sorted in ascending order for all participants. Next, the lower 27% and upper 27% percentiles were used to divide the participants into low and high groups, respectively. Independent samples *t*-tests were conducted to compare the scores of the two groups on each item, and the results revealed significant differences in the scores between the two groups on all items (*ps* < 0.01). Pearson correlation analyses were employed to explore the relationships between the scores of all participants on each questionnaire item and their corresponding subscale scores. The findings demonstrated significant correlations between the scores on all items and their respective subscale scores (*r* = 0.34~0.76, *ps* < 0.01).

The Cronbach’s *α* coefficients for the subscales of positive and negative aggression outcome expectancies were 0.85 and 0.94, respectively, indicating good internal consistency reliability of the questionnaire. To test the first hypothesis of this study, we conducted confirmatory factor analysis using Mplus7 software on AOEQ. Firstly, we employed the maximum likelihood method to extract two factors, namely positive and negative aggression outcome expectancies. The findings demonstrated that all item loadings surpassed 0.3, and the assignment of each item to its respective factor was consistent with the hypothesized model (see Table 1). Furthermore, the results revealed that the two-factor model of the AOEQ showed good fit indices ([34]; *χ*^2^/*df =* 1.30, CFI = 0.94, TLI = 0.94, RMSEA = 0.03, SRMR = 0.05), and there was no significant correlation between positive and negative aggression outcome expectancies (*r* = −0.08, *p* = 0.32). The results indicate that in the sample of college students from Study 1, aggression outcome expectancy exhibits a two-factor structure, and the two factors are relatively independent of each other.

## 3. Study 2

### 3.1. Participants

In Study 2, the appropriate sample size was decided, taking into account the results of an a priori power analysis using G*Power 3.1 [35]. Results showed that in a regression analysis with five predictors (sex, age, positive aggression outcome expectancy, negative aggression outcome expectancy, and moral disengagement), setting *α* = 0.05, effect size *f*^2^ = 0.15, adequate power = 0.95, the required sample size was ≥138.

Similar to Study 1, under the conditions of offering compensation and obtaining informed consent from participants, convenience sampling was used to distribute a total of 728 questionnaires collected through a combination of online and offline approaches. Additionally, in Study 2, we expanded the scope of data collection by including additional data sources from universities in Zhejiang and Sichuan Provinces. Study 2 employed the same criteria for data selection as Study 1, and data from questionnaires with inadequate quality were excluded, leaving a total of 677 valid questionnaires (with a valid response rate of 92.99%). In this sample, there were 322 male participants (constituting 47.56%) and 355 female participants (constituting 52.44%), with ages ranging from 17 to 24 years (*M*_age_ = 21.14, *SD*_age_ = 1.34). After confirming the questionnaire quality as acceptable, participants were provided with compensation.

### 3.2. Measurement

*Aggression Outcome Expectancy Questionnaire (AOEQ)*—The specific contents of the questionnaire are provided in Study 1. In Study 2, the Cronbach’s α coefficients for the positive and negative aggression outcome expectancy subscales were 0.90 and 0.95, respectively. The results of the confirmatory factor analysis indicated that the two-factor model demonstrated good fit indices (*χ*^2^/*df =* 2.83, CFI = 0.95, TLI = 0.94, RMSEA = 0.05, SRMR = 0.04). The results suggest that the questionnaire exhibits good reliability and construct validity.

*Civic Moral Disengagement Questionnaire (CMDQ)*—The CMDQ consists of 32 items (e.g., “Those who behave brutishly can only expect to be treated the same way by others”), rated on a 5-point Likert scale, where 1 represents “strongly disagree” and 5 represents “strongly agree” [36]. A higher overall average score indicates a higher level of moral disengagement. The CMDQ demonstrated good reliability and validity in the Chinese college student sample [37]. In Study 2, the CMDQ exhibited a high Cronbach’s *α* coefficient of 0.96, and the confirmatory factor analysis yielded satisfactory fit indices (*χ*^2^/*df =* 2.85, CFI = 0.91, TLI = 0.93, RMSEA = 0.04, SRMR = 0.05).

*Buss–Perry Aggression Questionnaire (BPAQ)*—Participants’ aggression in daily life was measured using the Physical and Verbal Aggression subscales of the Buss–Perry Aggression Questionnaire (BPAQ; [38]), which comprised a total of 14 items (e.g., “I get into fights a little more than the average person”). In Study 2, the Cronbach’s *α* coefficient for the Physical Aggression subscale was 0.91, and for the Verbal Aggression subscale was 0.84. After combining the two subscales, the overall Cronbach’s *α* coefficient was 0.90. The results of the confirmatory factor analysis indicated that the structure validity of the combined scale was satisfactory (*χ*^2^/*df =* 3.32, CFI = 0.90, TLI = 0.91, RMSEA = 0.05, SRMR = 0.06).

### 3.3. Statistical Analysis

We used SPSS25 for data analysis, including the common method deviation test, descriptive statistics, and Pearson’s correlation analysis. Furthermore, we assessed the hypothetical moderated mediation model using the Hayes PROCESS macro [39] with 5000 bootstrap samples and 95% confidence intervals. Prior to conducting the analysis, all quantitative variables were standardized to ensure comparability and mitigate the potential impacts of the scale differences among the variables.

### 3.4. Results

#### 3.4.1. Common Method Bias/Variance

To avoid potential common method bias, we conducted Herman’s single-factor test, which revealed that a single factor explained less than 40% of the variation (22.58%), indicating no common method bias.

#### 3.4.2. Descriptive Statistics and Correlation Analysis

In the college student sample, positive aggression outcome expectancy showed significant positive correlations with moral disengagement, aggression, and negative outcome expectancy. Moral disengagement was also significantly positively correlated with aggression. See Table 2 for details.

#### 3.4.3. Moderated Mediation Analysis

First, we employed model 4 from the Process Macro to examine the mediating role of moral disengagement in the relationship between positive outcome expectancy and aggression in the college student sample. After controlling for sex and age, the results revealed that positive outcome expectancy positively predicted aggression (*β* = 0.22, *SE* = 0.02, *p* < 0.001) and moral disengagement (*β* = 0.46, *SE* = 0.03, *p* < 0.001), and moral disengagement positively predicted aggression (*β* = 0.66, *SE* = 0.03, *p* < 0.001). The results indicate that the Bootstrap 95% confidence interval does not include 0 (95% CI = [0.25, 0.36]), with a mediation effect value of 0.30 for moral disengagement (accounting for 58.10% of the total effect). This suggests that the mediation effect of moral disengagement is significant, and positive outcome expectancy can indirectly predict aggression through moral disengagement.

Secondly, we incorporated negative outcome expectancy for aggression into the analysis and utilized model 8 to examine the moderated mediation model. With sex and age as control variables, the analysis demonstrated significant predictive effects of the interaction between negative outcome expectancy and positive outcome expectancy on both moral disengagement (*β* = −0.15, *SE* = 0.05, *p* < 0.001) and aggression (*β* = −0.17, *SE* = 0.04, *p* < 0.001). This indicates that negative outcome expectancy significantly moderates the relationships between positive outcome expectancy and both aggression and moral disengagement. Next, negative outcome expectancy was categorized into three levels: low (16th percentile), moderate (50th percentile), and high (84th percentile). We then conducted simple slope tests to explore the moderating effects of negative outcome expectancy on the two paths described above. See Table 3 for details.

**Figure 2:**
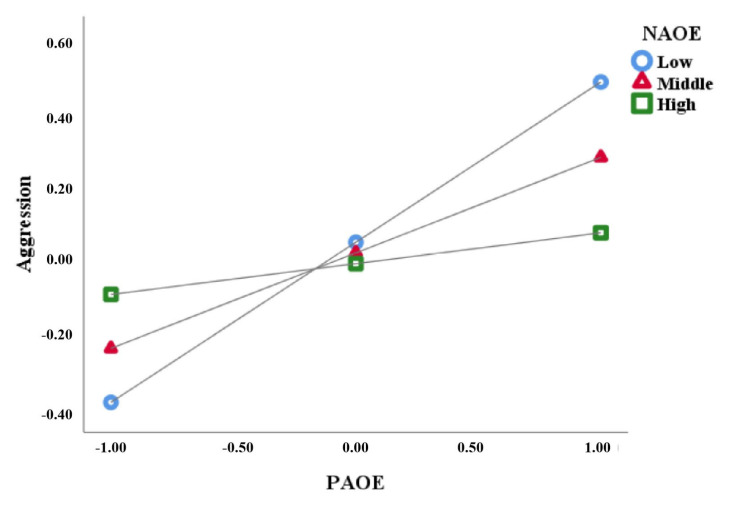
Moderating effect of NAOE on the relationship between PAOE and aggression. Note: PAOE = positive aggression outcome expectancy, NAOE = negative aggression outcome expectancy.

**Figure 3:**
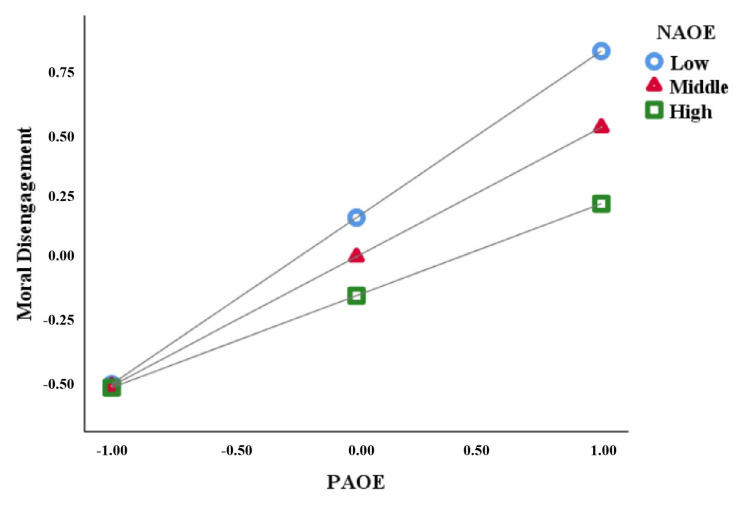
Moderating effect of NAOE on the relationship between PAOE and moral disengagement. Note: PAOE = positive aggression outcome expectancy, NAOE = negative aggression outcome expectancy.

The simple slope analysis indicated that as aggressive negative outcome expectancy levels increased, the predictive coefficients of positive outcome expectancy on aggression (*β*_high_ = 0.43, *β*_medium_ = 0.26, *β*_low_ = 0.08, *ps <* 0.05) and moral disengagement (*β*_high_ = 0.67, *β*_medium_ = 0.52, *β*_low_ = 0.37, *ps <* 0.001) decreased gradually. See Figure 2 and Figure 3.

## 4. Discussion

Firstly, the present study validated the two-factor structure of aggressive outcome expectancy, encompassing both positive and negative dimensions. Moreover, there is no significant correlation between the two (*r* = −0.08, *p* = 0.32; see Study 1), suggesting they are relatively independent. Next, we investigated the psychological mechanisms underlying the positive aggressive outcome expectancy predicting aggression based on the aggression motivation model [19,23,40], the aggression response evaluation–decision model [7], and the I^3^ theory [29]. The research findings revealed the mediating role of moral disengagement between positive outcome expectancy and aggression, as well as the moderating effect of negative outcome expectancy on the relationships between positive outcome expectancy and aggression, as well as positive outcome expectancy and moral disengagement.

### 4.1. Aggression Outcome Expectancy Comprises Two Factors: Positive and Negative Outcome Expectancies

Study 1 developed an Aggression Outcome Expectancy Questionnaire (AOEQ) for Chinese college students, and the results indicated that the questionnaire exhibited good item discrimination, internal consistency reliability, and construct validity. The cognitive assessment of behavior outcomes (gains or losses) before their execution plays a pivotal role in influencing decision-making [41,42], suggesting the existence of two fundamental dimensions in behavioral outcome expectancy: positive and negative. Findings from studies in substance addiction [43,44] and employment [45] support this perspective. However, in aggression research, while some scholars argue that aggression outcome expectancy encompasses both positive and negative dimensions that are relatively independent, direct empirical evidence is still lacking [6,7]. Study 1 provides direct evidence for the two-factor structure of aggression outcome expectancy, enriching existing aggression theories and facilitating further exploration of the cognitive mechanisms underlying aggression.

### 4.2. The Relationship between Positive Aggression Outcome Expectancy and Aggression Is Mediated by Moral Disengagement

The findings of Study 2 reveal that moral disengagement acts as a mediator between positive outcome expectancy and aggression. Specifically, positive outcome expectancy, as a typical aggressive approach factor, can promote college students’ aggressive behavior by enhancing their moral disengagement. According to moral disengagement theory [18] and the aggression motivation model [19,20,23,40], when individuals generate aggressive motives or intentions that do not align with their internalized moral standards, it leads to moral cognitive dissonance and an anticipation of moral sanctions (such as guilt). To avoid cognitive dissonance and moral inhibition, individuals may adopt a moral permission motivation, exemplified by moral disengagement, to facilitate the realization of aggressive intentions and promote the execution of aggression. In other words, the aggression motivation model presents an important psychological mechanism for aggression, characterized by the “aggressive approach motivation—moral permission motivation—aggression” pathway. The findings of Study 2 provide support for the propositions of the aggression motivation model and contribute to the further development of research on aggression-approach motivation. Moreover, the existing aggression cognitive theories (e.g., social learning theory, social information processing model) have primarily focused on the association between positive outcome expectancy and aggression. The mediating pathway discovered in Study 2 partly validates and broadens these theories, enhancing the theoretical underpinning of aggression research.

### 4.3. Negative Aggression Outcome Expectancy, Attenuates the Positive Predictive Effect of Positive Aggression Outcome Expectancy on Aggression

Study 2 revealed that negative outcome expectancy can moderate the relationships between positive outcome expectancy and aggression among college students. Specifically, negative outcome expectancy can weaken the positive predictive effect of positive outcome expectancy on aggression. This suggests that negative outcome expectancy can attenuate the influence of aggressive driving factors on aggression [13]. This might be because negative outcome expectancy weakens individuals’ perception of the value of aggression, thereby reducing the facilitating effects of positive outcome expectancy on aggressive intentions and behaviors.

### 4.4. Negative Aggression Outcome Expectancy, Attenuates the Positive Predictive Effect of Positive Aggression Outcome Expectancy on Moral Disengagement

Study 2 revealed that negative outcome expectancy can weaken the positive predictive effect of positive outcome expectancy on moral disengagement among college students. When negative outcome expectancy is at higher levels, it becomes difficult for positive outcome expectancy to evoke individuals’ aggressive intentions, and as a result, there is no need for moral disengagement to weaken or alleviate moral inhibition. Evidence from other research indirectly supports the aforementioned proposition, indicating that anticipated risks or negative outcomes can weaken the drivers of behavior, diminishing their facilitative effect on behavioral intentions [46,47] or perceived behavioral value [48]. This leads individuals to perceive the execution of the behavior as not worthwhile. For example, during the COVID-19 pandemic, the perceived risk of infection weakened the positive predictive effect of perceived behavioral control on travel intentions [47]. Habibi and Rasoolimanesh (2020) [48] also found that higher anticipated treatment costs attenuate the facilitating effect of healthcare quality on the perceived value of cross-border medical services.

## 5. Strengths and Applications

In summary, our findings have considerable theoretical and practical implications. Study 1 supported the two-factor structure of aggression outcome expectancy and provided a new measurement tool for investigating aggression outcome expectancy. Additionally, given the stable association between positive aggression outcome expectancy and aggression [9,10,12,13,15,25,26], this questionnaire can serve as a cognitive assessment tool for aggression. In practical applications, the findings of Study 2 suggest that higher education institutions should consider designing moral education programs specifically for college students to address their moral disengagement cognition and help them establish sound moral principles. Interventions should target the influence of aggression-driving factors on aggression, thus reducing aggressive behavior among college students. Study 2 enriched current aggression theories, offering empirical support for the aggression motivation model [19,20,23,40] and the I^3^ theory [29]. Furthermore, Study 2 revealed that negative outcome expectancy serves as a protective factor against aggression in college students. Therefore, higher education professionals should strengthen college students’ awareness of the negative consequences and risks of engaging in aggressive behavior, thereby diminishing the promoting effect of positive outcome expectancy on aggression. In conclusion, this research sheds further light on the motivational mechanisms of aggression from a cognitive perspective and provides valuable measurement tools and theoretical foundations for assessing, preventing, and intervening in aggression among college students.

## 6. Limitations

Despite the valuable contributions of the present study, there are still some limitations. Firstly, the Aggression Outcome Expectancy questionnaire was not assessed for test–retest reliability and criterion validity. Secondly, aggression outcome expectancy can be further divided into instrumental outcome expectancy (expectations of tangible benefits resulting from aggressive behavior, such as material rewards), emotional outcome expectancy, and social outcome expectancy [49,50]. Moreover, individuals’ expectancy for aggressive outcomes may vary across different forms of aggression [9,50] and relationship contexts [30,51]. This indicates that future research should further elaborate on the relationship between aggression outcome expectancy and aggression, identify the outcome expectancy that exerts prominent effects on aggressive behavior (salient belief) in distinct types of aggression, and explore the differences in their relationships across various contextual conditions (e.g., variations between campus and online bullying). Thirdly, this study utilized a cross-sectional design. For future research, longitudinal tracking and experimental approaches could be employed to explore the causal relationships between variables. Fourthly, social sociodemographic variables (such as family income) were not included as control variables in the research process. Lastly, the data collection method used in this study was convenience sampling. Therefore, further investigation is needed to determine whether the discovered mechanisms of aggression hold representativeness among Chinese college students. On the other hand, the generalizability of our findings to other populations or cultural backgrounds remains to be verified through replication studies.

## 7. Conclusions

In conclusion, among the college student participants, the current research discovered the following: (1) Aggression outcome expectancy demonstrates a two-factor structure comprising both positive and negative outcome expectations, and the two factors are relatively independent. (2) Moral disengagement mediated the relationship between positive aggression outcome expectancy and aggression. (3) Negative aggression outcome expectancy alleviated the positive prediction of positive aggression outcome expectancy on aggression. (4) Negative aggression outcome expectancy alleviated the positive prediction of positive aggression outcome expectancy on moral disengagement. Our study findings further illuminate the psychological mechanisms underlying aggression, offering valuable theoretical guidance for the prevention of and intervention in aggressive behavior.

## Figures and Tables

**Table 1:** The standardized factor loading of the Aggression Outcome Expectancy Questionnaire (*N* = 331).

PAOE		NAOE	
Items	Loading	Items	Loading
1	0.36	1	0.71
2	0.44	2	0.54
3	0.37	3	0.53
4	0.48	4	0.78
5	0.47	5	0.7
6	0.54	6	0.78
7	0.33	7	0.51
8	0.34	8	0.54
9	0.35	9	0.76
10	0.36	10	0.75
11	0.43	11	0.45
12	0.44	12	0.74
13	0.46	13	0.58
14	0.4	14	0.76
15	0.48	15	0.6

Note: PAOE = positive aggression outcome expectancy, NAOE = negative aggression outcome expectancy.

**Table 2:** The descriptive statistics for each variable (including the means and standard deviations) and correlations between variables (*N* = 677).

Variables	*M*	*SD*	1	2	3	4
1 PAOE	2.27	1.24	1			
2 Moral Disengagement	1.94	0.71	0.46 **	1		
3 Aggression	2.09	0.76	0.52 **	0.64 **	1	
4 NAOE	4.13	1.94	0.1 *	−0.04	0.05	1

Note: PAOE = positive aggression outcome expectancy, NAOE = negative aggression outcome expectancy, * *p* < 0.05, ** *p* < 0.01.

**Table 3:** The mediation–moderation model of the influence of positive aggressive outcome expectancy on aggression.

Variables	Model 1			Model 2			Model 3		
	Aggression			Moral Disengagement			Aggression		
	*β*	*t*	95% CI	*β*	*t*	95% CI	*β*	*t*	95% CI
Sex	0.07 *	2.18	[0.01, 0.14]	0.10 **	3.02	[0.04, 0.17]	0.01	0.47	[−0.04, 0.06]
Age	0.08 *	2.47	[0.02, 0.15]	0.20 ***	6.08	[0.14, 0.26]	−0.06	−2.37	[−0.11, −0.03]
PAOE	0.52 ***	15.78	[0.46, 0.58]	0.52 ***	13.96	[0.44, 0.59]	0.26 ***	8.31	[0.19, 0.32]
NAOE				−0.16 ***	−3.64	[−0.24, −0.07]	−0.03	−0.79	[−0.09, 0.04]
Moral Disengagement							0.65 ***	22.98	[0.6, 0.72]
PAOE * NAOE				−0.15 **	−3.06	[−0.25, −0.05]	−0.13 ***	−3.55	[−0.25, −0.08]
*R^2^*	0.29			0.28			0.61		
*F*	90.23 ***			52.89 ***			177.57 ***		

Note: Sex was dummy-coded (0 = female, 1 = male), and all regression coefficients were standardized. PAOE = positive aggression outcome expectancy, NAOE = negative aggression outcome expectancy, 95% CI = the 95% confidence interval, * *p* < 0.05, ** *p* < 0.01, *** *p* < 0.001.

## Data Availability

Upon reasonable request, the research data can be obtained by contacting the corresponding author.
